# Using social media for support and feedback by mental health service users: thematic analysis of a twitter conversation

**DOI:** 10.1186/s12888-015-0408-y

**Published:** 2015-02-19

**Authors:** Andrew Shepherd, Caroline Sanders, Michael Doyle, Jenny Shaw

**Affiliations:** Greater Manchester West Mental Health NHS Foundation Trust and University of Manchester, Manchester, UK; University of Manchester, Manchester, UK

**Keywords:** Service provision, Support, Mental disorder, Social media

## Abstract

**Background:**

Internet based social media websites represent a growing space for interpersonal interaction. Research has been conducted in relation to the potential role of social media in the support of individuals with physical health conditions. However, limited research exists exploring such utilisation by individuals with experience of mental health problems. It could be proposed that access to wider support networks and knowledge could be beneficial for all users, although this positive interpretation has been challenged. The present study focusses on a specific discussion as a case study to assess the role of the website www.twitter.com as a medium for interpersonal communication by individuals with experience of mental disorder and possible source of feedback to mental health service providers.

**Method:**

An electronic search was performed to identify material contributing to an online conversation entitled #dearmentalhealthprofessionals. Output from the search strategy was combined in such a way that repeated material was eliminated and all individual material anonymised. The remaining textual material was reviewed and combined in a thematic analysis to identify common themes of discussion.

**Results:**

515 unique communications were identified relating to the specified conversation. The majority of the material related to four overarching thematic headings: The impact of diagnosis on personal identity and as a facilitator for accessing care; Balance of power between professional and service user; Therapeutic relationship and developing professional communication; and Support provision through medication, crisis planning, service provision and the wider society. Remaining material was identified as being direct expression of thanks, self-referential in its content relating to the on-going conversation or providing a link to external resources and further discussion.

**Conclusions:**

The present study demonstrates the utility of online social media as both a discursive space in which individuals with experience of mental disorder may share information and develop understanding, and a medium of feedback to mental health service providers. Further research is required to establish potential individual benefit from the utilisation of such networks, its suitability as a means of service provision feedback and the potential role for, and user acceptability of, mental health service providers operating within the space.

## Background

Internet based social media websites such as Facebook (www.facebook.com) and Twitter (www.twitter.com) represent a growing facet of modern experience. These sites boast large numbers of users and their influence is increasingly being experienced in clinical practice [1]. Social media and forum websites have been examined as a resource for healthcare service users; for example Elwell and colleagues reviewed the role of internet based forums for adolescents living with cancer and found evidence of practical and emotional support being provided [2]. The role of Twitter has been reviewed in terms of the discussion of health screening procedures, such as mammography, again finding a mixture of information and personal narrative experience being described by users [3]. Internet based social media, such as Twitter, has also received attention for its potential in supporting social activism where its role can be seen in linking groups together and coordinating activity [4].

The role of the internet and social media as a resource for mental health service users as a possible means for reducing stigma and promoting help seeking behaviours [5] has been highlighted. However, little research has been conducted to explore this phenomenon. It could be hypothesised that the wider social network and experience available through internet resources will provide valuable support for people experiencing mental distress, as is the case with more traditional social networks. Cornwell and Laumann showed that an increase in social network size for older adults was associated with a decrease in symptom rating severity [6]. Similarly, examining the role of social networks in recovery from a variety of mental disorder experiences, Perry and Pescosolido observed that those who had access to networks consisting of firmer connections were found to have better functional recovery outcomes at follow up [7]. Similarly those with social networks containing members of a broadly pro-medical orientation experienced better outcomes. However, one study exploring the perception of social support and quality of life of internet users revealed a more complex picture where internet use for social and informational support was weakly correlated with an increase in perceived social support, but with a decrease in quality of life [8]. This finding may be reflecting a withdrawal from concrete social activity for increased engagement with electronic communication.

For the current project the website Twitter was used to allow a review of the utilisation of social media by mental health service users. The study therefore aimed to provide an initial exploration of the following: 1) The manner in which social media users with experience of mental disorder relate to each other and the social space during internet based interactions. 2) The potential role of resources such as Twitter for the provision of feedback on and engagement with mental health service user experience.

A qualitative content analysis approach was adopted in exploring these questions as it was believed that this would provide a pragmatic means of identifying salient themes displayed in the discourse between service users.

## Methods

The website www.twitter.com allows users to communicate in statements, *tweets*, of up to 140 characters in length. Users are identified by unique *user names* and can choose whether to reveal their real name to other users. Similarly, users may choose to reveal their geographical location through the website’s preference settings. Privacy settings for accounts are determined by individual users and range from open access accounts that can be identified through general internet searches to closed accounts, the outputs of which are only visible to other users registered as *followers*. Communications between groups of users can be facilitated through the use of *hashtags* where tweets are labeled such that other users can view contributions to conversations. For the purpose of this study a hashtag conversation - #dearmentalhealthprofessionals was identified. This conversation arose primarily over the weekend of the 10th - 11th August 2013 and was conducted in English, involving participants primarily from the United Kingdom and United States. The greatest flux of conversational material was generated over this weekend, although some minimal traffic continues through the hashtag on an on-going basis.

### Ethical approval

The research of internet based social media platforms potentially allows access to material relating to a large body of people, not all participants may be readily identifiable as individuals and seeking informed consent from each user can be complex. It is recognised that research on closed internet forums, available to specific registered users only, requires individual consent from all participants as the presence of “lurking” researchers may be viewed as invasive or hostile [9]. The role of consent to research based on publicly available material, such as that on www.twitter.com, is less clear, although previous researchers have argued that such material exists in the public domain with the proviso that prominent biographical details are excluded [10]. For the purpose of this research it was understood that users identifying with the conversation #dearmentalhealthprofessionals may have a past history of mental health difficulties and could be viewed as vulnerable research participants. However, it can also be argued that users, in agreeing to the terms and conditions of the website and then openly contributing to a public discussion, have accepted that they broadcast into the public domain and the role of research in such a space is not specifically precluded.

Ultimately the methods of this study were developed in accordance with ethical guidance published by the Association of Internet Researchers (www.aoir.org) so as to minimise the risk of individual distress through consideration of what might be considered a reasonable degree of personal privacy given the medium of communication. The protocol was reviewed and agreed by a University of Manchester research ethics committee.

### Search strategy

The native search engine provided at www.twitter.com was used in order to identify tweets containing the marker #dearmentalhealthprofessionals and occurring on the dates 10-11th August 2013. Identified tweets were copied as text and imported into an electronic spreadsheet (Numbers for Apple Mac OS X www.apple.com/mac/numbers/).

### Data analysis

The identified search material was reviewed allowing removal of any personally identifying or geographical material in order that that tweets were rendered anonymous. Sorting of the text allowed removal of duplicate material, known as *retweets*. Tweets were initially read and then re-read with an identifying code being applied to each tweet - this coding used phrasing from the source tweet in order to maintain close allegiance with original meaning. Tweets were then organised according to descriptive headings and overarching themes were identified. Coding was conducted initially by AS with independent review by other members of the research team - coding disagreements were resolved through discussion leading to refinement of the coding framework. Initial extensive and overlapping codes were reduced to three core categories through team discussion. Overarching themes were discussed and developed during research team meetings.

Writing was incorporated into the data analysis process such that iterative presentations of data and explanation of themes could be reviewed and discussed [11]. Representative quotations were identified during the writing process; material contradicting identified quotations and themes was specifically sought.

The possible role of researcher theoretical allegiance on the analysis process was addressed through discussion in team meetings. Research team members were drawn from a variety of theoretical orientations - psychiatry, mental health nursing and medical sociology.

## Results

The search strategy, following elimination of duplicate material, produced a total of 515 individual tweets. Descriptive coding and summary themes are represented alphabetically in Table 1, together with counts of tweets within each theme. The theme *Therapeutic relationship and developing professional communication* contained the greatest amount of material. The theme *Thanks* represented direct statements of thanks to individuals or clinical teams and is not discussed in any further detail. Self referential material refers to statements endorsing the hashtag and calling on others to participate. Linking out material represented links to other resources, primarily reflective blogs written by conversation participants, this material was not accessed in order to maintain confidentiality and minimal invasiveness for the research process.

**Table 1:** **Themes, descriptive headings and tweet counts**

**Theme**	**Descriptive heading**	**Count**
*The impact of diagnosis on personal identity and as a facilitator for accessing care*		*95*
	Care provision	13
	Individual	58
	Nature of diagnosis	14
	Overshadowing	2
	Role	4
	Stigma	4
*The balance of power between professional and service user*		*12*
	Individual power	1
	Political	1
	Professional	2
	Sharing	1
	Systemic	1
	Threatening	6
*Therapeutic relationship and developing professional communication*		*198*
	Burnout	5
	Carer communication	1
	Communication	37
	Compassion	22
	Critical	35
	GP	6
	Handover/sharing	6
	Learning	3
	Paramedic	2
	Police	3
	Professionalism	37
	Respect	10
	Risk	1
	Support	2
	Team working	1
	Understanding	26
	With client	1
*Support provision through medication, crisis planning, service provision and wider society*		*77*
	Accessing care	15
	Crisis	4
	Non-psychotropic	9
	Pharmacological	19
	Psychological	3
	Resources	13
	Service availability	9
	Society	5
Thanks		36
Self referential		75
Linking out		22
**Total unique Tweet count**		515

Individual themes are described below; while themes are presented as discrete, overlap of content exists between them.

### The impact of diagnosis on personal identity and as a facilitator for accessing care

Tweets within this theme addressed the issue of diagnosis in mental disorder from a variety of perspectives. The first descriptive group considered the role of diagnosis in allowing individuals to gain access to mental health care or to identify other means of potential support.

*“…accurate diagnosis enabled me to research, help plan my own recovery & find support groups.”*

This was combined with the consideration given to the role of diagnosis described by other participants - for example considering the role of diagnosis in allowing the individual to make sense of their experience within a specific framework, or allowing access to social benefit financial payments.

*“…’lets not put labels on your experience, but focus on the actual experience instead’ DOES NOT HELP! Dx pls!”* [sic]

This representation of diagnosis as having primacy over understanding of background experience was not reflected in other statements that focussed more on the nature of mental disorder diagnoses:

*“But many #DSM diagnoses are not mental disorders, they are social constructons.”* [sic]

*“Any given #DSM diagnosis says as much, if not more about the #DearMentalHealthProfessionals making the diagnosis as it does about the client”*

Participants also expressed concern relating to a loss of the sense of self for the individual following diagnosis; where behaviours and identity were subsumed within presumed illness states. The stigma that attended receiving a mental disorder diagnosis was commented upon and clinicians were called on to focus on the delivery of care in an appropriately holistic and humane manner.

*“…please don't assume every action, phrase, hair colour, dress choice is a symptom. I am not just a diagnosis.*”

*“…Avoid labelling- simplifying a person's problems to a word. Be aware of 'name-calling’…”*

*“…I am NOT JUST another nameless faceless #statistic. Nor am I the MERE end-result of a #corrupted system.”*

*“…I'm a person. Not a statistic. Not a number. I should be treated w/ respect and dignity.”*

*“…ban 'attention-seeking' & 'manipulative'. People still need help, even if been taught awkward ways of asking.”*

### The balance of power between professional and service user

This theme contained tweets which discussed the role of power, the ability to exert influence and control over a situation, and its reflection in the experience of mental distress. This perceived power could be held solely by individuals, shared between the service user and professional or abused by different parties.

*“…The forced treatment that was 'for my own good' did no good at all. It was harmful & left me traumatised.”*

*“INFORMED Choice #DearMentalHealthProfessionals why does this not happen routinely?”*

*“…making me terrified that you're going to commit me for suicide ideation does not promote sharing and openness”*

*“…don't use a patients leave request as blackmail to get them to do what you want”*

Through participation in this discussion individuals join a wider discourse that considers the balance of power between professional and service user. Within the physical health literature the sharing of decision making power has been described as the ‘pinnacle of patient centred care’ [12]. Within mental health practice a tension is still perceived however between the concepts of sharing power and more traditional models of care that ultimately consider risk and its control as paramount [13].

### Therapeutic relationship and developing professional communication

Tweets coded within this theme represented the greatest volume of material and reflected on various aspects of communication between professionals as well as professional attitudes while also highlighting the multitude of other agencies involved in the support of mental distress for example the police, primary care professionals and emergency medical services. The need to relate to individuals with compassion, particularly at times of crisis and to ensure clear lines of communication between professionals, service users and carers were emphasised.

*“…make sure at least one person knows wtf is going on as you pass patients from pillar to post.”*

*“…for all your/our training, humility is a key trait. We need to learn from and with those whom we serve.”*

*“Why do two GPs in the same practise give opposite advice? Who am I supposed to trust when I am so vulnerable?”*

*“Thank you for supporting me when I was caring for someone else. Carers also need cared for.”*

A large number of statements within this theme, coded as *critical*, were reflections of dissatisfaction with the care received. Aside from the negative valence of the content, these comments were difficult to interpret as they were often brief, or referred to specific events without detail. A number of conversation participants did however make statements that, while critical in nature, also clarified their cause for concern relating to the mental health, learning or professionalism of care providers; indicating perhaps an acknowledgement of work done in difficult circumstances yet requesting greater sympathy in addition to that already offered.

*“…You've always been overworked and undervalued, and it's getting worse. Pls recognise own burnout & depression”*

*“…if you've run out of compassion & just view us as a nuisance, please find another career & don't put it on us”*

*“…Most of you do an exceptional job in sometimes difficult circumstances. A few need to remember we're people.”*

### Support provision through medication, crisis planning, service provision and the wider society

The majority of material within this theme referred to concerns relating to an over reliance on medication as the primary treatment in response to mental distress.

*“…Don't become a pill-pusher Psychiatrist if you are truly interested in #mentalhealth and care about people”*

*“…I've known a few Drs try anti-psychotics, and every time then one pill has radically changed practice.”*

Also highlighted were concerns regarding side and withdrawal effects relating to psychotropic medication.

Other comments focussed on the role of psychotherapy - emphasising the importance of psychological service provision, but also bemoaning the limited availability of varying therapeutic modalities.

*“#DearMentalHealthProfessionals who are working to make #psychotherapy affordable: Thank you.”*

*“CBT is not the answer to everything”*

*“…#dearmentalhealthprofessionals Just because all your CMHT can offer is meds or CBT does NOT mean it is the most effective treatment for me.”*

A number of comments in this theme also highlighted the role of treatments that would be considered non-standard in terms of currently published clinical guidance. These comments perhaps represent an acceptance of such online forums as a space in which treatments beyond the mainstream may be discussed openly.

*“…Landmark discovery - RCT - Personalised approach to B Vit therapy for Schizophrenia.”*

The role of care planning was discussed, particularly the role of identifying crisis management plans, for example advance directives:

*“…It is terrifying to be pinned face down, even if it is on a crash mat! Please try other things first love me”*

*“One way #dearmentalhealthprofessionals helped me is by introducing me to crisis plans. Encouraged me to make my own as/when needed.”*

The nature of service provision was discussed focussing primarily on practical considerations relating to access to care, availability of services and resourcing. Concerns described included the perception of diagnostic criteria, for example specific weight restrictions, being used to approve access to care. Also particularly prevalent was the availability of services outside of the working, nine to five, day.

*“…just b/c i can articulate what's going on at a time doesn't mean i am too "functional" to need services”*

*“…criminal justice legal framework around patients should not affect their right of access to care.”*

*“…MH problems don't just happen Mon to Fri 9 am to 5 pm. They're always so much worse 'out of hours’…”*

*“…admit it if you've not got the resources to give me the help I need: better than pretending I don't need help”*

Some respondents also related their comments about mental health service support to the wider society, with participants suggesting that messages directed at mental health professionals could be reflected more generally, with an implicit reference to the experience of stigma:

*“…I know all you have is meds & talk. But pls acknowledge that sometimes the world needs changing, not me.”*

*“The #dearmentalhealthprofessionals tag is turning up some really good insights. Lots of it applies to society as a whole.”*

## Discussion

The presented study sought to use a case study conversation to explore the utilisation of online social media, in terms of its role as a social space for communication regarding the experience of mental disorder and as a means of understanding feedback on the experience of provided mental health care.

Much of the discussion identified by the search strategy was concrete in nature - discussing aspects of the care experience and practical means of addressing the concerns raised. Parallel to this however, was a discussion that focussed more on individual experience in mental disorder. For example considering the role and nature of mental disorder diagnoses; reflecting on diagnosis as a means of accessing care, or support, but also on the manner of their construction and development. Participants also considered the difficulties experienced within therapeutic relationships and their reflection in a wider societal context. That these topics received consideration within the conversation thread may represent the potential of social media platforms to allow discussion, reflection and sharing of experience by participants within the space provided. Although a limiting factor to this engagement is that potential participants, who may benefit from such discussion, may be excluded due to lack of internet access, therefore favouring the voices of those with sufficient privilege to access the space.

The nature of the Twitter platform, with a 140 character limit on communications, could be considered as reducing the potential for in-depth discussion. However the frequent presence of links to external resources, found through the reported search strategy, illustrate its potential strength as a central hub of discussion facilitating the coordination of groups of individuals with shared experience or interests. In this way, internet social media could be seen as providing a discursive resource that can provide access to greater numbers of people with shared experiences than may be expected through standard, more corporeal, social networks. This may allow for the development of larger support networks or for more direct communication between service users and professionals to be facilitated. Online social media could therefore provide a resource through which barriers to feedback, traditionally encountered by mental health services, can be overcome, as well as providing a medium which is readily amenable to data collection and analysis allowing rapid interpretation of feedback.

The phenomena of social media platforms being used as such a resource has been previously explored, primarily for resources relating to physical health care. Considering the role of internet based forums for discussion of HIV related issues, particularly disclosure of HIV status to sexual partners, Rier reported how such resources provided support in terms of information and discussion but how the material could often become deeply moralistic and inflammatory in nature [14]. This may therefore represent a risk in the nature of discussion that can occur through such fora. It is also unclear to what extent interaction through social media will lead to exposure to material that will challenge one’s natural point of view and lead to genuine reflection. Rier [14] commented on the scarcity of novel arguments within the content of material he observed. It is possible that participants may choose to engage with material that serves only to reinforce their currently held beliefs. This possibility is reinforced by an observation of social network construction within a free space as described by Centola and Van der Rijt [15]. In this study the authors observed that participants choosing contacts within a newly forming network preferentially selected individuals who closely matched them in terms of reported characteristics, potentially reducing the ability of networks to allow debate or encourage change.

There is also potential value for social media resources for health professionals to aid in the development of collaborative relationships with service users. Surveying a group of mental health practitioners in the United States, Deen and colleagues found a high prevalence of experience with social media usage among professionals but identified a confusion regarding the role of such resources in clinical practice. Some of their participants describing searching internet resources for information relating to their patients, but reflecting on the complex ethical issues surrounding this practice [1]. This professional involvement, which may potentially be viewed as invasive, would need careful consideration for its acceptability by mental health service users. This difficulty was recently highlighted through negative on-line response to the efforts of the charity Samaritans (www.samaritans.org) to introduce a service that would monitor social media feeds for material that may indicate suicidal ideation, which for some Twitter users represented an unacceptable invasion of privacy [16].

The themes identified within this study provide important messages to mental health providers - highlighting the features that mental health service users view as crucial to service provision including the need for understanding the implications of diagnosis and the importance of therapeutic relationships. These findings do not represent novel discussions as the role and importance of the therapeutic relationship within mental health care has been well described in the academic literature [17] and the difficulties with providing a service that is primarily biomedical in nature for example have been frequently commented upon [18,19] and continues to generate much debate [20]. Despite this lack of novelty the spontaneous nature of the discussion is perhaps remarkable - this conversation represented a previously unadvertised event emerging solely through user participation, its themes are representative of a wider discourse and serve to demonstrate the salience of such discussion within modern society and the role of social media in supporting and empowering mental health service users. As the greatest strength of the material observed in this study likely lies in its spontaneity, it represents a phenomenon that may be difficult to reproduce in a purposive manner. This may raise difficulties for mental health services seeking to exploit the media as a means of eliciting feedback - although the volume of material produced may indicate that this is an acceptable medium of communication for mental health service users.

### Limitations

The geographical reach of the analysed conversation is difficult to determine owing to the efforts made to preserve the anonymity of participants. The topics of discussion imply that the conversation was primarily held between respondents within the United Kingdom and United States. This wide reach may limit the specific applicability of findings - for example discussions relating to the costing of insurance and medical service provision is, currently, of limited interest to participants in the UK. As such it is possible that lessons for specific geographical regions may be lost within the broader conversation. Mental health providers looking to develop findings specific to their service may seek to directly approach individuals within their identified geographical region; a risk of such an approach however would be the loss of spontaneity and also possible restriction of dialogue through direct observation by professionals. Additionally the restriction of needing internet access in order to participate in the conversation may mean that it is not entirely representative for all mental health service users.

The analysis of this conversation is limited to being descriptive in nature owing to the nature of the subject material. As has been commented above some participants bypassed the 140 character limit through providing links to external resources. These materials were not examined in order to preserve anonymity but may provide a valuable resource for future research, with author consent. As the analysis strategy was primarily descriptive in nature the impact of author theoretical allegiance is likely minimal but was addressed and discussed during research supervision and through iterative draft writing in the representation of analysis and findings.

The search strategy adopted for this study sought to capture material generated on two days. The nature of the search engine available on the website www.twitter.com may limit claims of representativeness in the obtained material however. For example the search engine favours material that has demonstrated popularity, for example through number of endorsements by “re-tweets”, geographical biases are introduced through favouring material that is locationally close to the searcher, or emphasising material that contains references to other users within the searcher’s own network of contacts. Ultimately it can not be guaranteed that the search strategy was totally comprehensive in its reach. However for the purpose of this analysis it can be argued that the volume of material identified supports the validity of findings.

The precise identity of participants in this project can not be verified. However for the purpose of this study it is proposed that self-identification as a mental health service user, through participation in the online conversation, allows an initial exploration of the role of online social media as a social space to be made. This approach is analogous to other studies where large volumes of social media data are aggregated to develop information of use to clinical service provision [21]. The utilised search approach is unable to identify the role of those who may read the conversation but not participate directly in it. This group of individuals may gain benefit from such participation but this will be more difficult to identify and explore. The role of silent observer requires further exploration as studies, such as Rier [14], observed that the majority of forum participants occupied the role of a silent “lurker”.

Finally the methodology utilised in this study is unable to demonstrate behavioural change resulting from participation in this conversation by either mental health service users or professionals. Further work will be required to demonstrate such change.

## Conclusions

This study has outlined a role for resources, such as Twitter, in providing a discursive space and support for those with experiences of mental disorder. The methods used do not allow comment on the potential benefit or detriment from such use however.

Given the existence of conflicting evidence relating to possible benefits and harms from online social media use [8] further research focussing on exploration of the use of social media by mental health service users and any benefits gained is necessary. Additional work should include the exploration of potential roles for mental health service providers, and the acceptability of such a presence within this social space for all participants. The prevalence of external material linked to and referenced in the discussion also invites further consideration; exploration of such material may involve identification of the author however and this would require greater ethical consideration than was necessary for this project. The use of anonymous automated data mining techniques may represent a more appropriate approach in future research as they provide an anonymous means of identifying large volumes of material. Examples of such methods have previously been used to explore emotional material contained within suicide notes [22]. Social media appears to have a potentially rich role as feedback medium for mental health care service provision, more work is required to determine whether this strength can be generated on demand, in what manner such discussion could be controlled so as to generate pertinent feedback and how such feedback can be utilised alongside other feedback communication avenues. For example Greaves and colleagues have developed techniques through which patient comments in relation to care are aggregated and used as a means to detect poor clinical care [23].

In summary, this study has described the potential role of Twitter as a means of both service feedback and a space in which mental health can be openly discussed and considered from a variety of perspectives. The precise role that it occupies in the lives of those experiencing mental disorder and the possibilities, or appropriateness, of professional involvement in this space requires further research.
